# Environmental Factors Associated With Soil Prevalence of the Melioidosis Pathogen *Burkholderia pseudomallei*: A Longitudinal Seasonal Study From South West India

**DOI:** 10.3389/fmicb.2022.902996

**Published:** 2022-07-01

**Authors:** Tushar Shaw, Karoline Assig, Chaitanya Tellapragada, Gabriel E. Wagner, Madhu Choudhary, André Göhler, Vandana Kalwaje Eshwara, Ivo Steinmetz, Chiranjay Mukhopadhyay

**Affiliations:** ^1^Department of Microbiology, Kasturba Medical College, Manipal Academy of Higher Education, Manipal, India; ^2^Faculty of Life and Allied Health Sciences, Ramaiah University of Applied Sciences, Bengaluru, India; ^3^Diagnostic and Research Institute of Hygiene, Microbiology and Environmental Medicine, Medical University of Graz, Graz, Austria; ^4^Friedrich Loeffler Institute of Medical Microbiology, University Medicine Greifswald, Greifswald, Germany; ^5^Division of Clinical Microbiology, Department of Laboratory Medicine, Karolinska Institutet, Stockholm, Sweden; ^6^ICAR-Central Soil Salinity Research Institute (CSSRI), Karnal, India; ^7^German Federal Institute for Risk Assessment, Berlin, Germany; ^8^Centre for Antimicrobial Resistance and Education, Manipal Academy of Higher Education, Manipal, India; ^9^Centre for Emerging and Tropical Diseases, Manipal Academy of Higher Education, Manipal, India

**Keywords:** *Burkholderia pseudomallei*, melioidosis, South West India, environmental surveillance, soil, physicochemical factors, seasonal variation

## Abstract

Melioidosis is a seasonal infectious disease in tropical and subtropical areas caused by the soil bacterium *Burkholderia pseudomallei.* In many parts of the world, including South West India, most cases of human infections are reported during times of heavy rainfall, but the underlying causes of this phenomenon are not fully understood. India is among the countries with the highest predicted melioidosis burden globally, but there is very little information on the environmental distribution of *B. pseudomallei* and its determining factors. The present study aimed (i) to investigate the prevalence of *B. pseudomallei* in soil in South West India, (ii) determine geochemical factors associated with *B. pseudomallei* presence and (iii) look for potential seasonal patterns of *B. pseudomallei* soil abundance. Environmental samplings were performed in two regions during the monsoon and post-monsoon season and summer from July 2016 to November 2018. We applied direct quantitative real time PCR (qPCR) together with culture protocols to overcome the insufficient sensitivity of solely culture-based *B. pseudomallei* detection from soil. A total of 1,704 soil samples from 20 different agricultural sites were screened for the presence of *B. pseudomallei*. Direct qPCR detected *B. pseudomallei* in all 20 sites and in 30.2% (517/1,704) of all soil samples, whereas only two samples from two sites were culture-positive. *B. pseudomallei* DNA-positive samples were negatively associated with the concentration of iron, manganese and nitrogen in a binomial logistic regression model. The highest number of *B. pseudomallei-*positive samples (42.6%, *p* < 0.0001) and the highest *B. pseudomallei* loads in positive samples [median 4.45 × 10^3^ genome equivalents (GE)/g, *p* < 0.0001] were observed during the monsoon season and eventually declined to 18.9% and a median of 1.47 × 10^3^ GE/g in summer. In conclusion, our study from South West India shows a wide environmental distribution of *B. pseudomallei*, but also considerable differences in the abundance between sites and within single sites. Our results support the hypothesis that nutrient-depleted habitats promote the presence of *B. pseudomallei.* Most importantly, the highest *B. pseudomallei* abundance in soil is seen during the rainy season, when melioidosis cases occur.

## Introduction

Melioidosis is a potentially fatal infectious disease that is known to be highly endemic in Thailand and Northern Australia (Cheng and Currie, 2005; Hinjoy et al., 2018; Smith et al., 2018; Hantrakun et al., 2019). The disease is caused by the bacterium *Burkholderia pseudomallei*, listed as a Tier 1 select agent by the Centers for Disease Control (CDC), since it can be aerosolized and misused as bioweapon. *B. pseudomallei* is intrinsically resistant to many antibiotics (Schweizer, 2012). There is no vaccine available to date. The pathogen can be isolated from natural habitats, such as soil and surface water (Cheng and Currie, 2005; Trung et al., 2011; Kaestli et al., 2012; Lau et al., 2014; Baker et al., 2015; Jilani et al., 2016; Ribolzi et al., 2016; Dance et al., 2018). It infects humans through percutaneous inoculation, inhalational or ingestion routes, leading to protean clinical manifestations, ranging from mild localized infection to acute septicemia with multiple organ failure (Cheng and Currie, 2005). Some of the most common risk factors for melioidosis are diabetes mellitus, an age above 45 years, alcoholism, liver disease, chronic lung and kidney disease (Wiersinga et al., 2018). While an increasing number of case reports from different parts of the world, including China, South America, Africa and different parts of Asia, have been described in recent years (Rolim et al., 2005, 2018; Lin et al., 2016; Mukhopadhyay et al., 2018), a modeling study from 2016 predicted an enormous worldwide underreporting of the disease with an estimated global incidence of 165,000 cases and 89,000 deaths per year (Limmathurotsakul et al., 2016). The highest burden of the disease was predicted for India with about 52,000 cases and 31,425 (13,405 – 75,601) deaths annually (Limmathurotsakul et al., 2016). In sharp contrast to these figures, only 583 cases of melioidosis were reported in India from 1991 to 2016, of which 231 cases were reported from a single tertiary care center located in Manipal, Karnataka during 2006 to 2016 (Mukhopadhyay et al., 2018). This obvious discrepancy between prediction and diagnostic reality highlights the need for improved awareness of melioidosis in India, including an assessment regarding where potential risk areas of infection are located. However, as with other known or suspected melioidosis endemic areas, data from India on the environmental *B. pseudomallei* presence are limited and only a few relatively small studies have addressed this issue (Prakash et al., 2014; Peddayelachagiri et al., 2016; Chandrakar and Dias, 2017).

In addition to spatial clustering, an association of melioidosis cases with seasonal rainfall In addition to spatial clustering, an association of melioidosis cases with seasonal rainfall has been observed for a long time in known endemic regions including Northeast Thailand and Australia (Suputtamongkol et al., 1994; Currie and Jacups, 2003; Baker et al., 2011; Suebrasri et al., 2013; Liu et al., 2015). An association of cases with periods of heavy rain fall was also observed in India and in the tertiary care center in Manipal, Karnataka, where the majority were diagnosed during the monsoon season (Vidyalakshmi et al., 2012; Mukhopadhyay et al., 2018). However, the cause of this seasonal clustering remains uncertain. Periods of heavy rainfall and severe weather events might increase the likelihood of exposure to environmental *B. pseudomallei* and/or effect the environmental abundance of the pathogen.

Reliance on culture protocols to identify factors associated with the abundance of *B. pseudomallei* in the environment could be imprecise due to the low sensitivity of soil cultures. Previous studies have clearly shown that the current consensus methods for the culture (Limmathurotsakul et al., 2013) detection of *B. pseudomallei* lack sensitivity, leading to false-negative results (Trung et al., 2011; Gohler et al., 2017; Trinh et al., 2019). A study from Laos showed a higher detection rate of *B. pseudomallei* in enrichment cultures from soil samples by PCR when compared to *B. pseudomallei* growth in the respective subcultures (Dance et al., 2018), which is probably due to insufficient selectivity of the culture media used. Studies including soil from Vietnam and Thailand revealed that completely culture-independent direct quantitative real time PCR (qPCR) detection increased the rate of *B. pseudomallei*-positive soil samples significantly (Trung et al., 2011; Gohler et al., 2017).

Against this background, the present study (i) aimed to determine the prevalence of *B. pseudomallei* in the soil of melioidosis endemic regions in South West India by using direct qPCR together with culture protocols, (ii) investigate whether geochemical factors are associated with the presence of *B. pseudomallei* and (iii) test the hypothesis if there is an association between the seasonal incidence of human infections and the *B. pseudomallei* burden in soil.

## Materials and Methods

### Study Area

The study was conducted in in the southwestern coastal part of India in the districts of Udupi and Shimoga. Udupi is located directly on the seaside and is bordered on the northeast side by Shimoga district, that lies approximately 150 km inland. Udupi district in the state of Karnataka covers a total area of 3,582 km^2^ with a population density of approximately 300 inhabitants per square kilometer. Shimoga district covers 8,485 km^2^ area having about 200 inhabitants per square kilometer. The annual rainfall recorded in Udupi from January 2016 to January 2019 ranged between 3,763 and 4,423 mm and in Shimoga from 832 to 2,982 mm. Paddy is the most common crop cultivated in both regions. In 2016, 25.2% of the population in Shimoga and 15.7% in Udupi were involved in agricultural activity.

### Soil Sampling

During a pilot study in November 2014, and July and November 2015, 360 soil samples (78, 103, and 179, respectively) each of about 50 g were collected close to the home of melioidosis patients at eight different sites in the Udupi region at 10 and/or 30 cm depth from agricultural or formerly agricultural land. Samples were collected according to the consensus method (Limmathurotsakul et al., 2013). Three (S1, S2, S3) out of eight sites had been included in the main study, starting from July 2016 onward.

**FIGURE 1:**
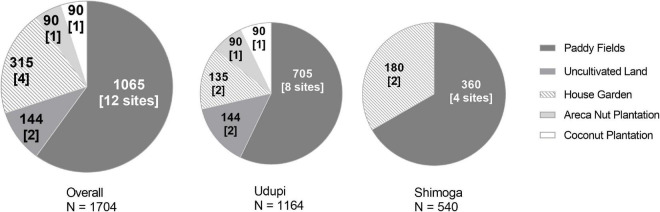
Number and field quality of soil samples collected for the detection of *Burkholderia pseudomallei.* Each sector of the circle stands for a respective type of field. Sample counts are given as bold numbers; the number of sites is written in brackets.

From July 2016 to January 2019, 81 culture-confirmed cases of melioidosis were diagnosed at Kasturba Medical College, Manipal, Karnataka. Among these 81 patients, eleven patients died. 65 (80.2%) patients had diabetes mellitus, 14 (17.3%) records of hypertension and six patients (7.45%) displayed renal dysfunctions. In 35 (43.2%) of patients, the infection manifested as localized abscesses, in eight (9.8%) patients with pulmonary involvements and in 38 (46.9%) patients were bacteremic. Patients were interviewed about their habits and activities that might have caused *B. pseudomallei* infections by environmental exposure. Thirty-eight (47%) patients from a total of 81 cases were either agriculturist labor workers, construction worker, or people known to have soil contact through recreational activities, such as gardening or walking barefoot. Among the 38 patients with a probable risk of exposure, 20 (52.6%) gave consent to collect soil samples from their field. Twenty fields were sampled, including 14 from Udupi and 6 from Shimoga district (Figure 1). According to the consensus guidelines, the grid sampling technique was followed. A Z pattern was used to collect the soil samples for fields of a size less than 5,000 sq. ft. Approximately 500 g of soil were collected at 30 cm depth from each sampling point using an auger (Limmathurotsakul et al., 2013). Sampling points were referenced with a global positioning system.

The locations of the fields and the characteristics assessed, such as the type of crop planted and the type of soil, are shown in Table 1. All sites were sampled thrice, once in each of three subsequent seasons, to address seasonal variations. A total of 1,704 samples were collected during the whole study period (Table 1). All soil samples were processed within 48 h of collection for the identification of *B. pseudomallei*. A total of 1,065 samples (62.5%) from 12 locations (Udupi: 60.57%, 705 samples; Shimoga: 66.67%, 360 samples) originated from paddy fields and 639 (37.5%) from the remaining eight locations, including house gardens, an arecanut plantation, a coconut plantation and from uncultivated land (Udupi: 39.43%, 459; Shimoga: 33.33%, 180 samples; Figure 1). In further analysis, all field categories except paddy were designated to the term “Others.”

**TABLE 1:** Characteristics of soil sampling sites, time of sampling and number of samples collected for the detection of *Burkholderia pseudomallei.*

Site	Type of crop	Soil type	Location	Samples Collected					
				Summer (March–May)	Month/Year	Monsoon (June–October)	Month/Year	Post Monsoon (November–February)	Month/Year
S1	Paddy	Sandy clay loam	Udupi	25	March 2017	25	July 2016	25	November 2016
S2	Uncultivated land	Red lateritic	Udupi	21	March 2017	32	July 2016	21	November 2016
S3	Uncultivated land	Sandy loam	Udupi	25	March 2017	20	July 2016	25	November 2016
S4	House garden	Lateritic	Udupi	15	April 2017	15	July 2017	15	November 2017
S5	Areca nut plantation	Sandy loam	Udupi	30	April 2017	30	July 2017	30	November 2017
S6	Paddy	Loam	Shimoga	30	March 2018	30	July 2017	30	November 2017
S7	House garden	Sandy loam	Shimoga	30	March 2018	30	July 2017	30	November 2017
S8	Paddy	Sand	Udupi	30	March 2018	30	July 2017	30	November 2017
S9	Paddy	Sand	Udupi	30	March 2018	30	July 2017	30	November 2017
S10	Paddy	Sandy loam	Udupi	30	March 2018	30	July 2018	30	November 2017
S11	House garden	Red lateritic	Shimoga	30	March 2018	30	July 2018	30	November 2017
S12	Paddy	Sandy loam	Shimoga	30	March 2018	30	July 2018	30	November 2017
S13	Paddy	Sandy clay loam	Udupi	30	March 2018	30	July 2018	30	November 2017
S14	Paddy	Sandy clay loam	Shimoga	30	March 2018	30	July 2018	30	November 2018
S15	House garden	Lateritic	Udupi	30	March 2018	30	July 2018	30	November 2018
S16	Paddy	Sandy loam	Udupi	30	March 2018	30	July 2018	30	November 2018
S17	Paddy	Sandy clay loam	Udupi	30	March 2018	30	July 2018	30	November 2018
S18	Coconut plantation	Red lateritic	Udupi	30	March 2018	30	July 2018	30	November 2018
S19	Paddy	Sandy loam	Udupi	30	March 2018	30	July 2018	30	November 2018
S20	Paddy	Sandy clay loam	Shimoga	30	March 2018	30	July 2018	30	November 2018
TOTAL				**566**		**572**		**566**	

### Soil Properties

For the physicochemical measurements, an equal number of qPCR positive and negative soil samples (60 samples each) was included. In each season, one qPCR positive and one negative sample was randomly selected from each field for the analysis of the physicochemical factors. The distance between the sampling points for physicochemical analysis ranged from 1 to 5 m. Samples were analyzed for acidity [pH], exchangeable sodium and potassium [ppm] (Jackson, 1959), available phosphor [ppm] (Olsen et al., 1954), manganese [mg/kg], total iron [g/kg], copper [mg/kg], available zinc [mg/kg] (Lindsay and Norvell, 1978), total nitrogen [mg/kg] (Eastoe and Pollard, 1950) and electrical conductivity (dS/m), as described previously (Jackson, 1959). An amount of 200 g of soil was air-dried and sieved to remove any debris prior to further analyses of the chemical factors. The soil types were classified according to the Standard Practice for Classification of Soils for Engineering Purposes (ASTM D. 2487 -06).

### Direct Soil DNA Extraction and PCR

In the pilot study between November 2014 and November 2015, DNA was extracted from 0.5 g of soil using the innuSPEED Soil DNA Kit (Analytik Jena, Germany). The DNA samples were analyzed by qPCR, targeting three different *B. pseudomallei*-specific gene sequences including the TTSS1 target (Gohler et al., 2017). Based on the positive signal of all three qPCR targets, selected soil samples were subjected to culture enrichment for the isolation of *B. pseudomallei* bacteria (see below).

In the main study starting in November 2016, the DNA was extracted from 0.1 g of soil from the samples included using an innuSPEED soil DNA kit (Analytik Jena, Germany), according to the manufacturer’s instructions. The DNA was eluted in 50 μl elution buffer and stored at −80°C until further testing. In a next step, the extracted DNA was subjected to a PCR with universal bacterial primers targeting the 16S gene to validate the general abundance of bacterial DNA in the samples (Lane, 1991). As all 1,704 DNA samples were positive for the 16S gene target, they were subsequently subjected to a qPCR targeting a *B. pseudomallei*-specific site of the TTSS1 gene (Novak et al., 2006). All primers and probes used in this study are listed in Supplementary Table S2. An individual standard curve of serially diluted genomic DNA of K96243 with determined concentrations (10^1^ to 10^6^ copies/μl) was generated for each single real-time PCR assay to quantify *B. pseudomallei* genome equivalents in the extracted DNA (Trung et al., 2011). The PCR assay was carried out in a Rotor Gene Q (Qiagen, Germany) thermocycler with an initial denaturation step at 95°C for 5 min, 45 cycles of denaturation at 95°C for 15 s and amplification at 60°C for 1 min.

### DNA Extraction From Enrichment Broth

An amount of 1 ml of the supernatant from the cultures incubated for 48 h in the crystal violet colistin broth (CVC-50) (Limmathurotsakul et al., 2013) was collected and centrifuged at 3,024 g for 2 min. The DNA was extracted from the pellet using an innuSPEED soil DNA kit (Analytik Jena, Germany). A amount of 4 μl DNA was subjected to qPCR targeting the TTSS1 gene of *B. pseudomallei* using the same primers and PCR conditions as mentioned above (Novak et al., 2006).

### Soil Culture Methods

Soil samples of the pilot study with a positive signal in all three *B. pseudomallei*-specific qPCR targets (Gohler et al., 2017) were cultivated according to standard procedures (Limmathurotsakul et al., 2013) and additionally in a two-step approach. A period of 48 h of incubation in Ashdown broth (Ashdown, 1979) were followed by a 96 h incubation in a minimal medium based on TBSS-C50 (Galimand and Dodin, 1982) with erythritol as a single carbon source. At this time, the erythritol-based medium was under development and differed from the version published later (Trinh et al., 2019) by the N source (0.1% ammonium chloride) and the addition of a diverse set of antibiotics (32 mg/L tobramycin, 50 mg/L colistin, 16 mg/L norfloxacin, 50 mg/L gentamicin, and 50 mg/L cycloheximide) (Goodyear et al., 2013). Enrichments were subcultured in serial dilutions on Ashdown agar. Colonies with a typical morphology were subjected to a colony PCR of a *B. pseudomallei*-specific TTSS1 gene sequence for the identification of putative *B. pseudomallei isolates* (Winstanley and Hart, 2000).

Soil cultures of samples collected during the main study from 2016 to 2018 were performed according to a consensus standard protocol using CVC-50 (Wuthiekanun et al., 1995; Limmathurotsakul et al., 2013) and a modified protocol using CVC-50 for the incubation of filters that were obtained from soil suspension filtration. Briefly, 100 g of soil was mixed with 100 ml sterile distilled water and allowed to settle overnight. A quantity of 1 ml of the supernatant was added to 9 ml of CVC-50 and incubated in static conditions at 37°C for 48 h. After incubation, 50 μl of the surface liquid was plated and incubated on Ashdown agar for 5 days at 37°C. An amount of 200 g of soil were mixed with 200 ml of distilled water and left overnight for the filtration method. A quantity of 100 ml of the supernatant from the soil-water mixture was filtered using Whatman No 1 filter. Following filtration, the filter paper was incubated statically in 20 ml of CVC-50 broth at 37°C for 48 h. After incubation, 50 μl of the CVC-50 broth was subcultured on Ashdown agar, as described previously.

### Identification of *B. pseudomallei* and Other *Burkholderia* spp.

Putative *B. pseudomallei* isolates were confirmed by a latex agglutination assay (Anuntagool et al., 2000) and a TTSS1 PCR (Novak et al., 2006). Subsequently, whole genome sequencing of the respective *B. pseudomallei* isolates was performed using bacterial DNA extracted with Qiagen DNA mini kit (Qiagen, Germany). The identification of other *Burkholderia* species was performed using matrix–assisted laser desorption/ionization method (MALDI-TOF, bioMerieux, France). Isolates with probability values of identification below 60% and more than one suggested species as a result of the MALDI-TOF analysis were additionally confirmed by *recA* PCR followed by sequencing of the amplified fragment (Payne et al., 2006).

### *B. pseudomallei* Phylogenetic Analysis

Illumina short reads of the environmental *B. pseudomallei* isolates from this study were submitted to the Sequence Read Archive under the NCBI accession ERR9146399 (IND_S3) an ERR9138530 (IND_S14). Whole genome sequencing data of *B. pseudomallei* strains were analyzed in SeqSphere (Ridom GmbH, Germany) using our previously published B. *pseudomallei* core genome MLST (cgMLST) scheme (Lichtenegger et al., 2021). A UPGMA tree and a minimum spanning tree were constructed based on the allelic profiles of the isolates. Columns with missing values for at least one sample were removed before analysis, resulting in 3635 targets/distance columns. Figures were created and annotated with Ridom SeqSphere.

### Statistical Analysis and Visualization of Data

The map of sampling locations was prepared with the Tableau Desktop test version. Visualization and statistical analyses of data were performed with GraphPad Prism software, version 8.2.1 and IBM SPSS Statistics, version 27. The Fisher’s exact test with Bonferroni correction was used to evaluate the significance of differences between the number of clinical cases per season and for the number of *B. pseudomallei* positive soil samples under respective conditions, for example, season. Load variations of the pathogen among soil samples with detectable C*_*T*_* values (positive samples) were analyzed with the Kruskal–Wallis test followed by Dunn’s test of multiple comparisons. The same test was applied for an explorative analysis of quantity deviations of physicochemical soil parameters in *B. pseudomallei* positive compared to negative soil samples. A multivariable binomial logistic regression model was constructed to examine the relationship between positivity and physicochemical soil parameters, such as iron, manganese, conductivity and others, to account for potential synergistic effects of different physicochemical parameters based on data of 60 *B. pseudomallei* positive and 60 negative samples. Predictors were selected manually and excluded stepwise to refit the model. Collinearity among geochemical factors was excluded prior to the model building process based on a correlation matrix including all geochemical parameters. The Hosmer–Lemeshow test was used to assess the goodness of fit of the model. Statistical significances with probability values (*p*) below 0.05 were considered significant and marked with starlets on top of a horizontal line above the data sets compared in the depictions (*p < 0.05, ^**^p < 0.01, ^***^p < 0.001, ^****^p < 0.0001).

### Ethics Statement

The study did not involve any human participants and was exempted from ethical review by the Ethics Committee, Kasturba Hospital, Manipal. However, permission and consent were obtained from private owners before sampling the site.

## Results

### Site Selection for the Detection of *B. pseudomallei* From Soil in the State of Karnataka

During some pilot samplings in 2014 and 2015, we analyzed 360 samples from eight different fields in the Udupi region. The qPCR targeting three *B. pseudomallei*-specific genes (Gohler et al., 2017) revealed 216 PCR positive soil samples with at least one of three targets out of 360 samples. *B. pseudomallei* was isolated from one sample from site S3. This high qPCR positivity rate prompted us to address a seasonal pattern of *B. pseudomallei* prevalence in soil and to investigate the influence of other ecological factors in a systematic environmental survey.

**FIGURE 2:**
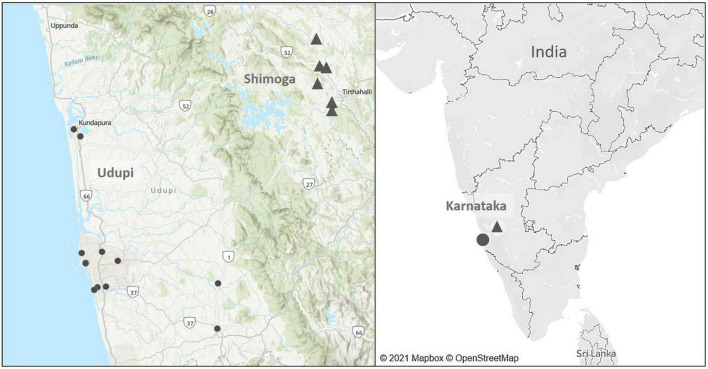
Map of soil sampling locations in the main soil surveillance study from July 2016 to January 2019. Black dots mark the sampling sites located in Udupi (14 fields) and triangles represent sites in Shimoga (six fields).

The selection of soil sampling sites was based on the geographical origin of culture-confirmed melioidosis patients diagnosed at Kasturba Medical College, Manipal, Karnataka from July 2016 to January 2019 (Figure 2). Among 81 patients, 50 lived in Udupi, 18 in Uttara Kannada, eleven in Shimoga, one in Chitradurga and another single patient came from the Chikmagalur district. The number of cases diagnosed during the monsoon (75%; 61/81) differed significantly (*p* < 0.001) from the number of cases post-monsoon (13.6%; 11/81) and during the summer (11.1%; 9/81).

As most patients from Udupi and Shimoga provided consent, soil samples were collected from 14 sites in Udupi and 6 sites in Shimoga during the monsoon, post monsoon and summer season from July 2016 to January 2019 (Figure 2). Sampling sites were selected according to a potential history of environmental exposure of patients due to occupation or lifestyle habits. Rainfall data of Udupi and Shimoga are given in the Supplementary Figure 1.

### Detection of *B. pseudomallei* in Soil Samples During Various Seasons Using Molecular and Cultural Methods

**FIGURE 3:**
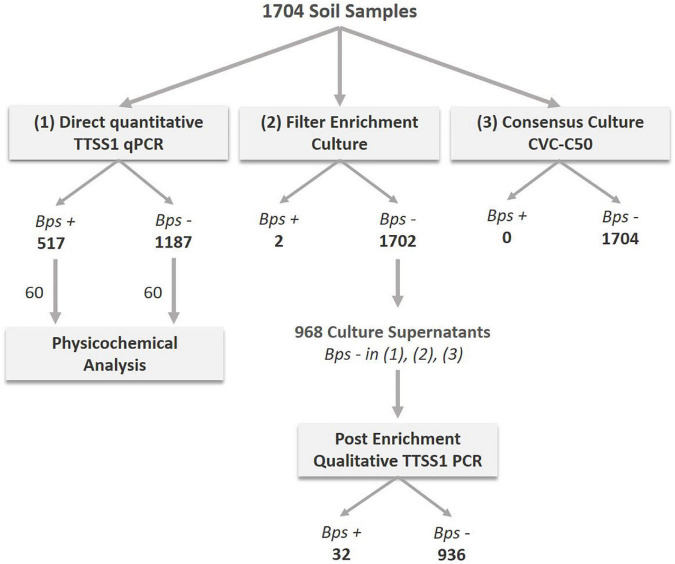
Analyses procedure of soil samples collected close to diagnosed melioidosis cases from July 2016 to January 2019. A total of 1,704 samples were processed and analyzed by direct TTSS1-qPCR (1) and cultivated using two different culture approaches: the consensus method (3) and a drainage filter enrichment (2). The number of *B. pseudomallei* positive (*Bps*+) and negative (*Bps–*) samples detected are given as bold numbers below the method. From July 2017 onward, 968 samples that were negative by direct qPCR and in the culture methods (2) and (3) were selected for an additional molecular analysis of the filter enrichment broth of method (2). A total of 60 TTSS1-qPCR positive and 60 negative soil samples were subjected to a physicochemical analysis.

The high number of melioidosis cases during the monsoon made us hypothesize that this seasonal disease pattern corresponds to a seasonal variation of *B. pseudomallei* abundance in soil. In other words, higher pathogen loads in soil during monsoon could be one among several factors leading to the higher melioidosis incidence during this time of the year. Therefore, 1,704 soil samples collected at a depth of 30 cm from 20 different fields during the monsoon, post-monsoon and dry season were subjected to direct TTSS1 qPCR, the consensus standard culture method in CVC-50 (Wuthiekanun et al., 1995; Limmathurotsakul et al., 2013) and a culture enrichment of filters obtained from soil water drainage. Starting from July 2017, samples negative in all three detection methods (968) were additionally subjected to a qualitative PCR of the filter enrichment broth (Figure 3). A total of 517 samples (30.3%) of 1,704 soils collected were tested positive for *B. pseudomallei* by direct qPCR and two of those samples were culture positive. Among 968 soil samples, which were negative by direct qPCR and in subcultures of both enrichment methods, 32 (3.3%) samples showed at least a positive signal in a qualitative TTSS1 PCR in the enrichment broth.

### Significant Differences in the Positivity Rate of Soil Samples Across the Seasons

**FIGURE 4:**
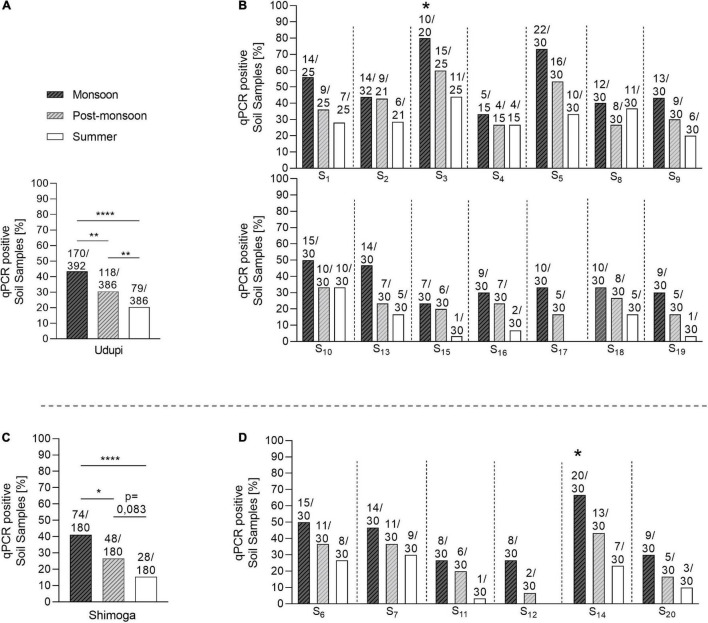
Seasonal prevalence of *B. pseudomallei* qPCR-positive soil samples from Udupi and Shimoga districts. **(A)** *B. pseudomallei* positive detected samples collected in Udupi in different seasons. **(B)** Percentage of positive soil samples detected in Udupi separated by sites. **(C)** *B. pseudomallei* positive samples collected in Shimoga in different seasons. **(D)** Percentage of positive detected soil samples in Shimoga separated by sites. The two starlets above the bars of site S3 and S14 mark culture positive sampling sites (Fisher’s exact test: *p < 0.05, **p < 0.01, ****p < 0.0001).

Samples collected during the monsoon season showed the highest positivity (244/572; 42.93%) that differed significantly from the positivity rates observed in the post-monsoon (166/566; 29.3%; *p* < 0.0001) and summer season (107/566; 18.9%; *p* < 0.0001). Furthermore, a significantly higher amount of positive samples was detected in the post-monsoon compared to the summer season (*p* < 0.001). This seasonal dependency of the overall positivity was also observed when broken down to the regions sampled, Udupi and Shimoga (Figures 4A,C), and was consistent over the single sites of both regions (Figures 4B,D). There was no significant difference in the positivity rate between Udupi and Shimoga in any season.

### Seasonal Variations in *B. pseudomallei* Loads in Soil Samples

**FIGURE 5:**
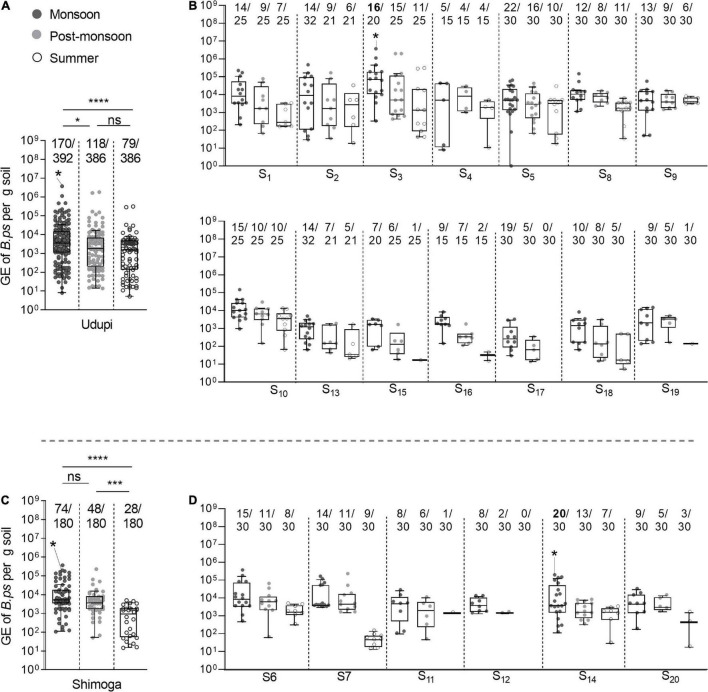
Seasonal differences of *B. pseudomallei* burden in soil samples. **(A,B)** The *B. pseudomallei* burden of qPCR positive samples throughout the seasons of Udupi and its single sites (Kruskal–Wallis, *p < 0.05, ***p < 0.001, ****p < 0.0001). **(C,D)** Burden of *B. pseudomallei* in positive detected soil samples collected at Shimoga and its sites. Samples that became positive in subsequent enrichment culture are marked with an asterisk in the data set. The measured molecular load of the culture positive soil samples was 3.76 × 10^6^ genome equivalents in the sample of site S3 and 1.95 × 10^5^ genome equivalent for site S14. Boxes line values from the 25th to 75th percentiles of a data set; the vertical line in a box plot marks the median value. Whiskers from the lower and upper quartile represent 1.5 times the interquartile range.

We next analyzed the *B. pseudomallei* loads in those 517 positive samples and tested for significant variations among the three seasons. The median value of those 517 positive soil samples was 3.3 × 10^3^ (IQR: 4.98 × 10^2^ – 9.5 × 10^3^) *B. pseudomallei* genome equivalents per gram of soil. The median of 4.45 × 10^3^ (IQR: 1.49 × 10^3^ – 1.68 × 10^4^) was significantly higher in the monsoon season compared to those in the post-monsoon (2.37 × 10^3^; IQR: 4.02 × 10^2^ – 7.6 × 10^3^, *p* = 0.0089) and summer season (1.47 × 10^3^; IQR: 8.16 × 10^1^ – 3.53 × 10^3^, *p* < 0.0001; Figure 5A), and still higher in the post-monsoon compared to the summer season (*p* = 0.0036). Considered separately for Udupi (Figures 5A,B) and Shimoga (Figure 5C,D), the differences between loads detected in the monsoon season in Udupi (3.47 × 10^3^; IQR: 1.13 × 10^3^ – 1.55 × 10^4^) and Shimoga (5.02 × 10^3^; IQR: 3.29 × 10^3^ – 1.78 × 10^4^) compared to the summer season in Udupi (1.55 × 10^3^; IQR: 1.35 × 10^2^ – 4.99 × 10^3^) and Shimoga (9.18 × 10^2^; IQR: 5.37 × 10^1^ – 1.80 × 10^3^) were again highly significant (*p* = 0.0001 and *p* < 0.0001, respectively). Comparing the loads in soil in both regions, no significant differences in the *B. pseudomallei* burden between Udupi and Shimoga was detectable. In summary, the positivity rate and loads between both regions decreased significantly from rainy season to dry season with no significance between the regions.

### Influence of Field Type on *B. pseudomallei* Detection

**FIGURE 6:**
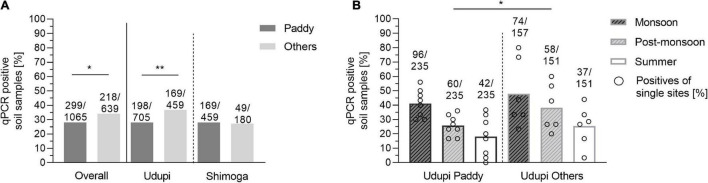
Positivity rate of *B. pseudomallei* soil samples of paddy and other locations. **(A)** Positivity of soil samples based on direct qPCR in paddy and other fields depicted for all 1,704 samples and separated for Udupi and Shimoga. **(B)** *B. pseudomallei* positivity rate of soil samples from paddy fields (left) and other locations of Udupi (right). Dots in the bars represent the percentage of positive samples for single sites (Fisher’s exact test: *p < 0.05, **p < 0.01).

We then compared the proportion of positive soil samples from diverse agricultural sites (218/639; 34.27%) with the paddy samples (299/1065, 28.08%) (Figure 6A). The positivity rate of other soil samples was significantly higher than the rate of paddy fields (*p* = 0.045) with an odds ratio of 1.33 (95% CI: 1.08 – 1.63). We repeated the analysis for each region separately: Only Udupi displayed a significant difference between the field types (*p* < 0.01) caused by a higher proportion of positive samples in the post-monsoon season (*p* = 0.046). In order to demonstrate the variance among the other sites compared to paddy, Figure 6B shows the positivity of the single sites and the overall positivity of samples from Udupi per season. When data of the culture positive site S3, uncultivated grass land, were excluded from the analysis, significant differences in positivity between other locations and paddy fields could no longer be detected.

Overall, we did not find a significantly higher rate of positive samples from paddy fields compared to other locations. Non-paddy sites varied more strongly in their positivity rate compared to paddy fields.

**FIGURE 7:**
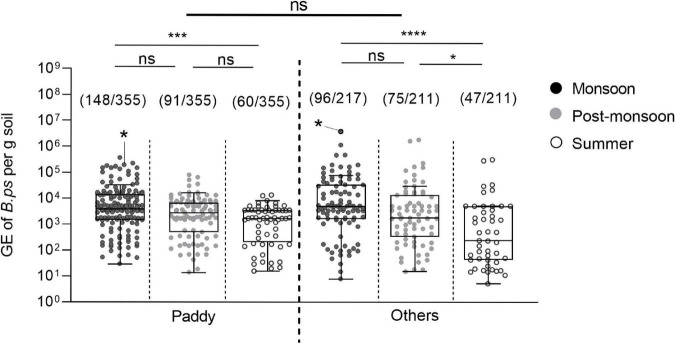
Seasonal difference in the burden of *B. pseudomallei* positive soil samples in paddy and other field types. Dot plots show *B. pseudomallei* genome equivalents (GE) per gram of soil detected in paddy and other fields sampled (Kruskal–Wallis; ns, not significant; *p < 0.05, ***p < 0.001, ****p < 0.0001). Samples that became positive in subsequent enrichment culture are marked with an asterisk in the data set. The measured molecular load of the culture positive soil samples was 1.95 × 10^5^ genome equivalent for the paddy site S14 and 3.76 × 10^6^ genome equivalents in the uncultivated site S3. Boxes line values from the 25th to 75th percentiles of a data set; the vertical line in a box plot marks the median value. The whiskers from the lower and upper quartile represent 1.5 times the interquartile range.

We also compared the *B. pseudomallei* burden between paddy and other locations and found no significant difference in the bacterial load. The seasonal *B. pseudomallei* loads of both field categories were significantly higher in the monsoon compared to the summer season (Figure 7).

### Physicochemical Properties of Soil in *B. pseudomallei* Positive and Negative Soil Samples

**FIGURE 8:**
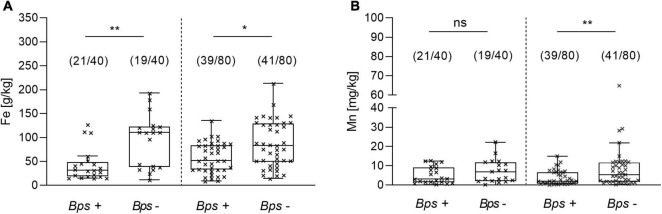
Difference in the iron and manganese concentration between *B. pseudomallei* positive (Bps+) and negative (Bps–) soil samples. Concentration of **(A)** iron in the monsoon (left side) and dry season (right site) and of **(B)** manganese. The term ‘dry season’ refers to the post-monsoon and summer season (Kruskal–Wallis, Dunn’s test; ns, not significant; *p < 0.05, **p < 0.01). Boxes line values from the 25th to 75th percentiles of a data set; the vertical line in a box plot marks the median value. The whiskers from the lower and upper quartile represent 1.5 times the interquartile range.

In a next step, we addressed the physicochemical properties associated to the abundance of *B. pseudomallei* in 120 soil samples, of which 60 direct qPCR-positive and 60 direct qPCR-negative were analyzed. Among all evaluated parameters including conductivity, pH, nitrogen, potassium, sodium, iron, manganese and others, significant differences in the univariate analysis were only observed in the median concentrations of manganese and iron between the positive and negative samples. Low iron concentration was associated with the PCR positivity of *B. pseudomallei* in the soil in both monsoon (*p* = 0.0017) and dry seasons (*p* = 0.02), which includes post-monsoon and summer (Figure 8A). A low concentration of manganese was related to the presence of *B. pseudomallei* in the dry season only (*p* = 0.0146) (Figure 8B).

A multivariate logistic regression model including the data of the 120 samples analyzed for their physicochemical properties revealed a negative association of *B. pseudomallei* with the presence of iron (OR, 0.980; 95% CI, 0.97 – 0.99; *p* = 0.000313), manganese (OR, 0.920; 95% CI, 0.85 – 1.0; *p* = 0.053) and nitrogen (OR, 0.993; 95% CI, 0.99 – 1.0; *p* = 0.028) in the soil samples analyzed. The model (*p* < 0.001, Nagelkerke’s *R*^2^ = 0.374) had an overall accuracy in classification of 77.5%, with a specificity of 73.3% and a sensitivity of 81.7%. No correlation among the independent metric variables was found.

### Isolation of *B. pseudomallei* Strains From Soil Samples During Various Seasons

Overall, we cultured three *B. pseudomallei* strains, two from site S3 (July 2015 and July 2016) and another one from site S14 (July 2018) by using the filter enrichment culture. The consensus method did not yield any *B. pseudomallei* isolate from any of the soil samples. A total of 1591 (93.7%) soil sample cultures led to the extraction of bacteria that were oxidase-positive, Gram-negative and grew in the presence of colistin and gentamicin. All isolates with a colony morphology similar to *B. pseudomallei* were tested using a latex agglutination assay and, if indicated, tested by TTSS1-PCR. The most common among other *Burkholderia* species isolated was *Burkholderia cepacia* (56.2%), followed by *Burkholderia vietnamensis* (30%), *Burkholderia multivorans* (12%), *Burkholderia cenocepacia* (5%), and *Burkholderia* sp. (7%) and other oxidase positive, gentamicin- and colistin-resistant organisms (3%).

### Genomic Analysis and Phylogenetic Relatedness of Environmental *B. pseudomallei*

**FIGURE 9:**
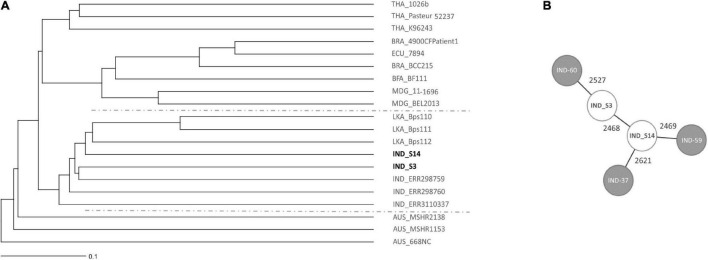
Core genome MLST (cgMLST)-based genomic comparison of the isolated Indian *B. pseudomallei* strains with a diverse, global collection of *B. pseudomallei* strains. **(A)** The UPGMA tree shows clustering of soil isolates from this study with other *B. pseudomallei* isolates from different parts of the world. The line in the lower left corner illustrates 0.1% cGLMST column difference (% of core genome target differences). The geographic origin of strains is given as country code prior to the strain name (THA Thailand, BRA Brazil, ECU Ecuador, BFA Burkina Faso, MDG Madagascar, IND India, LKA Sri Lanka, AUS Australia). **(B)** A cgMLST-based minimum spanning tree illustrates the genetic diversity of Indian isolates. Isolate names are given as short forms: IND-60 refers to ERR298760, IND-59 to ERR298759 and IND-37 to RR3110337. The numbers on the connecting lines refer to the number of allele differences. Strains included with accession numbers of respective databases are listed in Supplementary Table 1.

The multi locus sequence type of both *B. pseudomallei* isolates from site S3 were identical, therefore, only one isolate was subsequently sequenced together with the isolate from site S14. Whole genome sequencing demonstrates that the two environmental *B. pseudomallei* strains isolated cluster close to previously deposited Indian isolates in a tree of representative global isolates (Figure 9A). Other isolates within this clade originate from Sri Lanka. It is noteworthy that there is a great genetic diversity even within the Indian isolates, as shown by the minimum spanning tree (Figure 9B). The *B. pseudomallei* strains isolated during this study will be subjected to a detailed genetic analysis in a future study.

## Discussion

India has been predicted to be the country with the highest melioidosis burden globally, not least because of the possible environmental suitability for *B. pseudomallei* (Limmathurotsakul et al., 2016). On a global scale, multiple variables, such as rainfall, temperature, acrisol soil types and anthropogenic activities, were shown to be associated with the presence of *B. pseudomallei*. Larger data sets on the spacious dissemination and abundance of *B. pseudomallei* in India and its ecological drivers are not available. This is also true for the states of Kerala as well as Karnataka and Tamil Nadu, where most of the cases of melioidosis have been recorded (Mukhopadhyay et al., 2018). The environmental presence of *B. pseudomallei* in the coastal area of Tamil Nadu was documented in a study by Prakash et al. (2014), who described the isolation of four *B. pseudomallei* strains out of 45 paddy soil samples. Peddayelachagiri et al. (2016) aimed to examine the prevalence of *B. pseudomallei* and near-neighbor species in Malabar coastal region of Kerala and isolated five *B. pseudomallei* from thirty screened rubber plantation soil samples. A study from the coastal area of Karnataka reported the isolation of a single *B. pseudomallei* strain from 150 soil samples (Chandrakar and Dias, 2017). This limited number of *B. pseudomallei* culture-positive environmental samples obtained by using enrichment cultures is not surprising, as the restricted sensitivity of culture methods for environmental samples has been well documented in a number of studies, including soil from Thailand, Vietnam, and Laos (Trung et al., 2011; Gohler et al., 2017; Dance et al., 2018; Trinh et al., 2019). The reasons are ranging from insufficient selectivity of the standard media used (Wuthiekanun et al., 1995), to insufficiently validated incubation parameters and the possible existence of viable but non-culturable bacterial cells (Giagnoni et al., 2018). Moreover, enrichment cultures are not suitable to obtain quantitative data on the *B. pseudomallei* environmental load in single samples. Consequently, it is difficult to assess the true environmental pathogen distribution in melioidosis endemic areas.

As in other parts of the world melioidosis cases reported in South West India are also strongly correlated with times of heavy rainfall (Suputtamongkol et al., 1994; Currie and Jacups, 2003; Chou et al., 2007; Currie et al., 2010; Vidyalakshmi et al., 2012; Liu et al., 2015; Mukhopadhyay et al., 2018). Although this association with rainy seasons is a hallmark of melioidosis, the underlying causes remain to be determined. It is conceivable that the correlation observed could be the result of a higher chance of human exposure to the pathogen through aerosols, contaminated surface water and/or mud but also an increased pathogen load in the environment, a possibly altered physiological state of the bacterium leading to a higher infectivity, or a combination of those factors. The quantitative determination of *B. pseudomallei* in its environmental habitats during the various seasons is a prerequisite to investigate possible causes for the seasonal disease pattern. This point was addressed in a comprehensive study by Chen et al. (2014), in which *B. pseudomallei* concentrations in ambient air were quantified by qPCR. *B. pseudomallei* qPCR positive air samples were detected only during the typhoon season, but not in a reference season. Furthermore, *B. pseudomallei* DNA concentrations in ambient air were positively associated with rainfall days. The authors hypothesized that heavy rains and winds favor the inhalation of *B. pseudomallei*, which might be derived from soil surfaces (Chen et al., 2014).

In a number of studies, the seasonal variability of *B. pseudomallei* in soil samples was addressed by culture methods determining the proportion of *B. pseudomallei*- positive samples, quantitative bacterial loads were not determined (Baker et al., 2011; Suebrasri et al., 2013; Chandrakar and Dias, 2017; Swe et al., 2021). However, contradictory results were obtained, with some studies reporting higher rates of *B. pseudomallei*-positive samples during the dry season (Wuthiekanun et al., 1995), whereas others found higher isolation rates during the wet season (Suebrasri et al., 2013; Swe et al., 2021). By using a semi-quantitative culture method, a study from Laos found higher *B. pseudomallei* isolation rates in a rice field at 60 and 90 cm compared to 5 and 30 cm, however, no clear seasonal pattern of *B. pseudomallei* counts could be detected (Manivanh et al., 2017).

Against this background, we decided to systematically quantify the concentration of *B*. *pseudomallei* in soil during various seasons in order to test the hypothesis that seasonal variations in the soil load of this pathogen are linked to the seasonal appearance of the disease. Since it is now well documented that the molecular detection of *B. pseudomallei* in soil samples increases the rate of detection significantly (Kaestli et al., 2007; Trung et al., 2011; Gohler et al., 2017; Dance et al., 2018; Trinh et al., 2019) and *B. pseudomallei* DNA can be quantified directly from soil by using a TTSS1 qPCR (Trung et al., 2011; Gohler et al., 2017), we decided to apply this molecular approach together with culture methods. The TTSS1 qPCR (Novak et al., 2006) has been extensively used by others to detect *B. pseudomallei* in various habitats (Kaestli et al., 2007; Baker et al., 2011; Chen et al., 2014; Knappik et al., 2015; Zimmermann et al., 2018). Moreover, a rigorous validation of the TTSS1 qPCR across large *in silico* datasets (Price et al., 2012) and isolate collections confirmed 100% specificity (Trung et al., 2011; Price et al., 2012). Our *B. pseudomallei* detection in the main study relied on the single target TTSS1 qPCR, but not the use of three PCR targets, including TTSS1 as we did in the pilot study. A limitation of this resource-saving single target TTSS1 qPCR approach of our main study, is the potential underestimation of some samples with very low DNA amounts, since those samples might not be reliably detected by a single PCR assay (Gohler et al., 2017). Soil sampling at 30 cm depth was chosen, since this sampling depth has been applied in numerous studies (Wuthiekanun et al., 2005; Kaestli et al., 2007, 2009; Limmathurotsakul et al., 2010; Trung et al., 2011; Hantrakun et al., 2016; Manivanh et al., 2017; Dance et al., 2018; Saengnill et al., 2020; Swe et al., 2021) and the bacterial load at this depth is less likely to be influenced by short term environmental influences such as, e.g., UV light compared to soil surface. Still a depth in the range of 30 cm is accessible during agriculture activities such as plowing and cultivating soil leading to potential human exposition.

Our analysis of 1,704 soil samples collected from 20 different sites from two regions during the various seasons revealed not only the highest proportion of *B. pseudomallei-*positive samples during the monsoon (Figure 4), but also the highest *B. pseudomallei* loads in positive samples (Figure 5) as determined by direct qPCR. It is noteworthy that our qPCR approach also revealed a high diversity within single locations in either season. In other words, a significant percentage of samples from sites containing *B. pseudomallei*-positive samples with various pathogen loads were also *B. pseudomallei*-negative.

Future sampling strategies should take into account that soil is an extremely heterogeneous habitat, in which different niches exist at a hundreds of-micrometers or an even smaller scale (Vos et al., 2013). These local microenvironments might differ by unique biotic and abiotic parameters, such as temperature, pH, salinity, water, nutrient availability as well as microorganism diversity and abundance. The smaller and more delimited the collected samples will be for future analyses of soil parameters and *B. pseudomallei* loads, the closer we will get to the actual living environment of this pathogen. Importantly, the seasonal variation in *B. pseudomallei* abundance and the heterogeneity within single sites was consistent among different agriculture sites (Figures 6, 7). Our results indicate that, irrespective of the agricultural characteristics, monsoonal weather conditions lead to changed soil environments resulting in an increased *B. pseudomallei* concentration (Figures 5, 7). It is of note that there were no higher pathogen loads in paddy fields compared to a diverse set of other locations, including house gardens and other plantations, during all seasons. The only two samples which became culture-positive were collected from a grassland in Udupi and a paddy field in Shimoga during the monsoon and showed remarkably high loads by direct qPCR (Figure 5). Whole genome sequencing of those isolates and subsequent cgMLST (Lichtenegger et al., 2021) revealed clustering in the same group with other Indian isolates and strains from Sri Lanka, but also showed remarkable genetic difference between each other (Figure 9).

We also evaluated various physicochemical factors in a multivariable logistic regression analysis and found the concentrations of iron, manganese and nitrogen in a reverse association with the presence of *B. pseudomallei.* So far, environmental studies addressing soil factors which might have an impact on *B. pseudomallei* presence have been based on *B. pseudomallei* detection by enrichment cultures (Baker et al., 2011; Suebrasri et al., 2013; Chen et al., 2014; Ngamsang et al., 2015; Hantrakun et al., 2016; Swe et al., 2021). In a large environmental survey from Thailand, lower levels of organic matter, of phosphorus, potassium, calcium, magnesium, iron and salinity were correlated with the presence of *B. pseudomallei* in paddy soil samples (Hantrakun et al., 2016). Several additional studies also observed lower iron concentrations (Baker et al., 2015; Manivanh et al., 2017; Swe et al., 2021) and a low content of organic matter associated with *B. pseudomallei* presence (Baker et al., 2015; Ngamsang et al., 2015; Hantrakun et al., 2016; Manivanh et al., 2017). By comparing soil parameters of paddy field samples in Southern Iran with those of non-paddy soil samples, higher levels of available iron, manganese and organic carbon were detected in paddy soil (Azadi et al., 2021). These nutrient concentrations decreased with increasing soil depth (Azadi et al., 2021). Findings of higher numbers of *B. pseudomallei* in deeper soil horizons below 30 cm (Manivanh et al., 2017) strengthen the hypothesis that low nutrient levels correlate with the abundance of *B. pseudomallei*. Recently, Chewapreecha et al. (2022) suggested genetic adaptation of *B. pseudomallei* to nutrient depletion during evolution based on their extensive genomic and transcriptomic analyses.

However, contrary observations on the role of iron and the abundance of organic material have also been made (Wuthiekanun et al., 1995; Inglis and Sagripanti, 2006; Palasatien et al., 2008; Kaestli et al., 2009; Draper et al., 2010; Wang-Ngarm et al., 2014; Musa et al., 2016). These contrasting results may be attributed to the limitation of the culture method, foremost the unreliable classification of soils in “*B. pseudomallei* positive” and “*B. pseudomallei* negative” and have to be validated by further geochemical analysis of endemic sites.

Our finding of lower nitrogen, iron and manganese levels in *B. pseudomallei*-positive soil is in line with the assumption that sites containing less nutrients are more likely to be associated with the presence of *B. pseudomallei* (Figure 8). Nutrient-depleted environments might reduce the burden of other microbial soil inhabitants. Less competitive conditions would decrease the overall antagonism from inhibitory saprophytes in the presence of *B. pseudomallei* and/or opens selective nutritional and environmental niches for *B. pseudomallei*. Iron and manganese are essential micronutrients for bacteria and plant growth (Schmidt et al., 2016) and a restricted availability in soil might lead to a diminished rhizosphere and plant growth with a direct effect on the bacterial community.

High input of organic matter into the soil, its degradation and flooding of paddy fields (Azadi et al., 2021) promotes an oxygen-deficient environment. As a consequence, reduction of alternative electron acceptors will gain in importance and soluble Fe^2+^ and Mn^2+^ concentrations in the soil water will consequently increase. Additionally, fermentation processes will reduce soil water pH, again increasing the solubility of both ions. Although *B. pseudomallei* is capable of nitrate respiration under anaerobic conditions, higher cell densities are reached under aerobic conditions (Hamad et al., 2011). Therefore, not just competitive bacterial flora but also oxygen limitation, leading to a reduced *B. pseudomallei* and higher Fe^2+^ and Mn^2+^ concentrations might account for the observed negative correlation.

The dynamics and conditions in soil, like oxygen availability, salinity, the bacterial neighborhood, the nutrient offer, which enable *B. pseudomallei* to occupy its environmental niche, are complex and remain to be clarified. Small-scale analysis are an important prerequisite to gain a reliable understanding of associated parameters in the immediate environment of the bacteria. In conclusion, our study with soil samples derived from very different agricultural sites in two regions in South West India revealed a wide dissemination of *B. pseudomallei* with a considerable heterogeneity in the proportion of positive samples and pathogen load between sites and within single sites. Our results strengthen the hypothesis that nutrient-depleted habitats favor the presence of *B. pseudomallei.* Furthermore, we clearly demonstrate a correlation of *B. pseudomallei* abundance in soil and the occurrence of melioidosis in rainy seasons.

## Data Availability Statement

The datasets presented in this study can be found in online repositories. The names of the repository/repositories and accession number, PRJEB51514 can be found in the article/Supplementary Material.

## Author Contributions

TS, IS, and CM conceived the study. TS collected patients’ data and performed soil sampling. TS, CT, KA, AG, and VE participated in microbiological laboratory analysis. MC performed geochemical analysis. GW performed phylogenetic analyses. TS, KA, CM, and IS analyzed the overall data. KA and TS drafted the figures. KA performed the statistical analysis. TS, KA, and IS wrote the first draft. All authors reviewed the draft and approved the final version of the manuscript.

## Conflict of Interest

The authors declare that the research was conducted in the absence of any commercial or financial relationships that could be construed as a potential conflict of interest.

## Publisher’s Note

All claims expressed in this article are solely those of the authors and do not necessarily represent those of their affiliated organizations, or those of the publisher, the editors and the reviewers. Any product that may be evaluated in this article, or claim that may be made by its manufacturer, is not guaranteed or endorsed by the publisher.

## Supplementary Material

Supplementary Figure 1 Monthly rainfall provided by the India Meteorological Department and seasonal occurrence of melioidosis cases in Shimoga region **(A)** from 2016 to 2018. **(B)** Monthly rainfall and seasonal occurrence of melioidosis cases in Udupi region.
