# Supplementary figures and images for: Environmental Factors Associated With Soil Prevalence of the Melioidosis Pathogen Burkholderia pseudomallei: A Longitudinal Seasonal Study From South West India
Source: Front Microbiol. 2022 Jul 1;13:902996. doi: 10.3389/fmicb.2022.902996 (PMC9283100; doi:10.3389/fmicb.2022.902996)

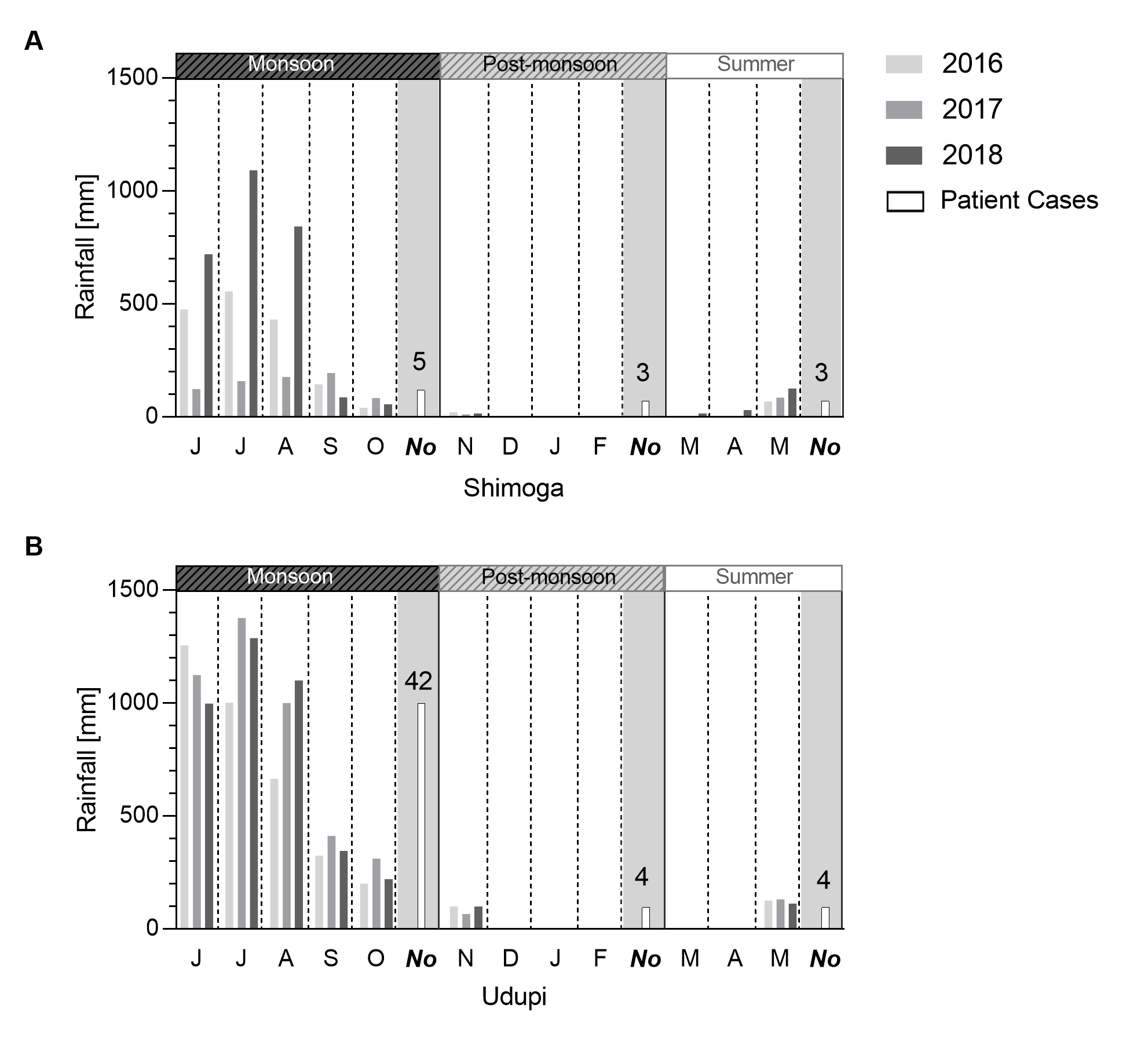

Supplement: Supplementary Figure 1 — Monthly rainfall provided by the India Meteorological Department and seasonal occurrence of melioidosis cases in Shimoga region (A) from 2016 to 2018. (B) Monthly rainfall and seasonal occurrence of melioidosis cases in Udupi region. [file Image_1.tif]
