# Supplementary material for: Environmental Factors Associated With Soil Prevalence of the Melioidosis Pathogen Burkholderia pseudomallei: A Longitudinal Seasonal Study From South West India
Source: Front Microbiol. 2022 Jul 1;13:902996. doi: 10.3389/fmicb.2022.902996 (PMC9283100; doi:10.3389/fmicb.2022.902996)
Supplement: Supplementary file 2 [file Table_1.docx]

| STRAIN NAME | FIG. 9 NOMECLATURE | NCBI or ENA ACCESSION NUMBER | ORIGIN |
| --- | --- | --- | --- |
| K96243 | THA_ K96243 | BX571965.1,BX571966.1  NC_006350 NC_006351 | Thailand |
| Pasteur 52237 | THA_ Pasteur52237 | JPNT00000000 | Thailand |
| 1026b BGR | THA_1026b | CP008834 CP008835 | Thailand |
| BCC215 | BRA_BCC215 | ABBR00000000 | Brasil |
| 4900CFPatient1 | BRA_4900 | NZ_ARZE00000000 | Brasilien |
| 7894 | ECU_7894 | CP009535 CP009536 | Ecuador |
| BF111 | BFA_BF111 | SRR3145395 | Burkina Faso |
| BEL2013 MSHR7966 | BDG_ BEL2013 | SRR3145396 | Madagaskar |
| 11-1696 | MDG_111696 | SRR3145393 | Madagaskar |
| IND_S3 | IND_S3 | ERR9146399 | India |
| IND_S14 | IND_S14 | ERR9138530 | India |
| H054640145 | IND-59 | ERR298759 | India |
| G9709 | IND-37 | ERR311037 | India |
| H103360117 | IND-60 | ERR298760 | India |
| BPs112 | LKA_Bps112 | CP037975, CP037976 | Sri Lanka |
| BPs110 | LKA_Bps110 | CP036451, CP036452 | Sri Lanka |
| BPs111 | LKA_Bps111 | CP036453, CP036454 | Sri Lanka |
| MSHR2138 | AUS_ MSHR2138 | JRFM00000000 | Australia |
| MSHR1153 | AUS_ MSHR1153 | CP009271 CP009272 | Australia |
| 668 | AUS_668NC | NC_009074 NC_009075 | Australia |

**Table S1: Strains mentioned in Figure 9 with respective database accession numbers.**
