# Supplementary material for: Environmental Factors Associated With Soil Prevalence of the Melioidosis Pathogen Burkholderia pseudomallei: A Longitudinal Seasonal Study From South West India
Source: Front Microbiol. 2022 Jul 1;13:902996. doi: 10.3389/fmicb.2022.902996 (PMC9283100; doi:10.3389/fmicb.2022.902996)
Supplement: Supplementary file 3 [file Table_2.docx]

| **Primers and probes** | **Sequence 5′-3′** | **Target** | **Source** | **Application** |
| --- | --- | --- | --- | --- |
| 27F  1429 R | AGAGTTTGATCCTGGCTCAG  GRTACCTTGTTACGACTT | 16S rRNA gene | (Lane, 1991) | qPCR from soil DNA for general bacterial abundance |
| BpTT4176 forward  BpTT4290 reverse | CGTCTCTATACTGTCGAGCAATCG  CGTGCACACCGGTCAGTATC | TTSS1 gene | (Novak et al., 2006) | *B.pseudomallei -*  specific qPCR from soil DNA and for strain verification |
| BpTT4208 probe | FAM-CCGGAATCTGGATCACCACCACTTTCC-BHQ1 | TTSS1 gene | (Novak et al., 2006) | *B.pseudomallei -* specific qPCR from soil DNA |
| BPSS0087-Lfw  BPSS0087-Brev  BPSS0087-P2 | GAATGCGTGCGCGAGCA  CTCGGCCGGTCCGGAAT  HEX-AGTCGTACGCAGCGCGCG-BHQ2 | Coding region of BPSS0087 | (Gohler et al., 2017) | *B.pseudomallei -*  specific qPCR from soil DNA |
| BPSS0745-Afw  BPSS0745-Fsrev  BPSS0745-P2 | GCGAGCAAATCCGTCTCTCA  ATGCCAGGGCACATGGCTA  FAM-ATCATTCAGGCGGGTGCCGT-BHQ1 | Coding region of BPSS0745 | (Gohler et al., 2017) | *B.pseudomallei -*  specific qPCR from soil DNA |

**Table S2: Oligonucleotides used in this study**

Gohler, A., Trung, T.T., Hopf, V., Kohler, C., Hartleib, J., Wuthiekanun, V., et al. (2017). Multitarget Quantitative PCR Improves Detection and Predicts Cultivability of the Pathogen *Burkholderia pseudomallei*. *Appl Environ Microbiol* 83(8)**,** e03212-03216. doi: 10.1128/AEM.03212-16.

Lane, D.J.S., E.; Goodfellow M. (1991). 16S/23S rRNA sequencing. *Nucleic Acid Techniques in Bacterial Systematic***,** 115-175.

Novak, R.T., Glass, M.B., Gee, J.E., Gal, D., Mayo, M.J., Currie, B.J., et al. (2006). Development and evaluation of a real-time PCR assay targeting the type III secretion system of *Burkholderia pseudomallei*. *J Clin Microbiol* 44(1)**,** 85-90. doi: 10.1128/JCM.44.1.85-90.2006.
